# Seasonality of parasitic and saprotrophic zoosporic fungi: linking sequence data to ecological traits

**DOI:** 10.1038/s41396-022-01267-y

**Published:** 2022-06-28

**Authors:** Silke Van den Wyngaert, Lars Ganzert, Kensuke Seto, Keilor Rojas-Jimenez, Ramsy Agha, Stella A. Berger, Jason Woodhouse, Judit Padisak, Christian Wurzbacher, Maiko Kagami, Hans-Peter Grossart

**Affiliations:** 1 Department of Plankton and Microbial Ecology, Leibniz Institute of Freshwater Ecology and Inland Fisheries (IGB), Zur alten Fischerhütte 2, 16775 Stechlin, Germany; 2 GFZ German Research Centre for Geosciences, Section Geomicrobiology, Telegrafenberg, 14473 Potsdam, Germany; 3 Faculty of Environment and Information Sciences, Yokohama National University, Tokiwadai 79-7, Hodogayaku, Yokohama, Kanagawa 240-8501 Japan; 4 Department of Ecology and Evolutionary Biology, University of Michigan, Ann Arbor, 48109 MI USA; 5 Escuela de Biología, Universidad de Costa Rica, 11501 San José, Costa Rica; 6 Department of Ecosystem Research, Leibniz Institute of Freshwater Ecology and Inland Fisheries (IGB), Müggelseedamm 301, 12587 Berlin, Germany; 7 Research Group of Limnology, Centre of Natural Sciences, University of Pannonia, Egyetem u. 10, 8200 Veszprém, Hungary; 8 Chair of Urban Water Systems Engineering, Technical University of Munich, Am Coulombwall 3, 85748 Garching, Germany; 9 Institute of Biochemistry and Biology, Potsdam University, Maulbeerallee 2, 14469 Potsdam, Germany; 10 Present Address: Department of Biology, University of Turku, Vesilinnantie 5, 20014 Turku, Finland; 11 Present Address: Marbio, UiT- The Arctic University of Norway, Sykehusveien 23, 9019 Tromsø, Norway

**Keywords:** Fungal ecology, Limnology

## Abstract

Zoosporic fungi of the phylum Chytridiomycota (chytrids) regularly dominate pelagic fungal communities in freshwater and marine environments. Their lifestyles range from obligate parasites to saprophytes. Yet, linking the scarce available sequence data to specific ecological traits or their host ranges constitutes currently a major challenge. We combined 28 S rRNA gene amplicon sequencing with targeted isolation and sequencing approaches, along with cross-infection assays and analysis of chytrid infection prevalence to obtain new insights into chytrid diversity, ecology, and seasonal dynamics in a temperate lake. Parasitic phytoplankton-chytrid and saprotrophic pollen-chytrid interactions made up the majority of zoosporic fungal reads. We explicitly demonstrate the recurrent dominance of parasitic chytrids during frequent diatom blooms and saprotrophic chytrids during pollen rains. Distinct temporal dynamics of diatom-specific parasitic clades suggest mechanisms of coexistence based on niche differentiation and competitive strategies. The molecular and ecological information on chytrids generated in this study will aid further exploration of their spatial and temporal distribution patterns worldwide. To fully exploit the power of environmental sequencing for studies on chytrid ecology and evolution, we emphasize the need to intensify current isolation efforts of chytrids and integrate taxonomic and autecological data into long-term studies and experiments.

## Introduction

Recent advances in sequencing technologies have revealed that fungi are ubiquitous and highly diverse in aquatic ecosystems [1, 2]. Yet, a substantial fraction of aquatic “dark matter” fungi, especially the early diverging lineages, has not been described [3]. Zoosporic fungi of the phylum Chytridiomycota (chytrids), regularly dominate pelagic communities in freshwater and marine environments [4–6]. Chytrids encompass a wide range of taxa with a continuum of consumer strategies spanning from strict saprotrophs to obligate parasites [7, 8]. As such, chytrids are decomposers of autochthonous and allochthonous organic matter such as zooplankton exuviae and pollen grains [9] and lethal parasites of phytoplankton [8, 10, 11]. The integration of chytrids in the PEG (plankton ecology group) model [12, 13] exemplifies the emerging recognition of chytrids as ecological and evolutionary drivers of phytoplankton bloom dynamics. Chytrids can suppress the development of phytoplankton blooms [14–16], selective chytrid parasitism can alter interspecific competition, affecting phytoplankton coexistence and succession [14, 17] and, by imposing negative frequency-dependent selection (e.g., “killing the winner” [18]), chytrid parasites maintain and promote genetic diversity in phytoplankton populations [19, 20]. Furthermore, chytrids efficiently siphon carbon and nitrogen from the photosynthetic host, bypassing the microbial loop (i.e., fungal shunt [21]), which is further transferred to zooplankton through the consumption of chytrid zoospores (i.e., mycoloop [22]). By this, chytrids modify microbial interactions, enhance herbivory [23–25] and accelerate carbon transfer to higher trophic levels in pelagic food webs.

Despite recent advances, we are still far from comprehensively characterizing the phylogenetic and ecological diversity of chytrids. Although 18S and 28S rRNA gene sequencing approaches have been applied to unearth chytrid diversity [26, 27], our current knowledge on the diversity, especially of phytoplankton parasites, is almost exclusively based on >100 years of morphology-based identification [7]. The scarcity of reference chytrid sequences in databases creates difficulties in linking chytrid sequences to specific ecological traits or their host ranges.

To overcome these limitations, we aimed at improving the linkage between chytrid sequence diversity and consumer-resource interactions by studying their seasonal dynamics in a well-studied lake ecosystem. We combined isolation approaches including 1) direct cultivation, 2) single-cell isolation, and 3) in situ baiting to target phytoplankton parasites and saprotrophic pollen-degrading chytrids. Cultivation enables detailed morphological and molecular studies on all chytrid life stages, while experimental cross-infection assays provide insights into their host range and specificity [28, 29]. Yet, cultivation is difficult and time-consuming, which can arguably underestimate diversity because not all chytrids can grow under the given laboratory conditions and phytoplankton hosts available. This limitation can be partially overcome by single-cell isolation, i.e., manual isolation and subsequent sequencing of single infected phytoplankton colonies/cells or pollen grains [28, 30]. For higher throughput and a greater coverage of diversity compared to manual cell picking, we applied an in situ baiting approach combined with amplicon sequencing to target and amplify chytrids associated with pollen.

These targeted approaches allowed us to establish a taxonomic and ecological annotated library compiling information on sequence, morphology, and host/substrate ranges. We applied this reference library to an amplicon-based high-throughput sequencing (HTS) dataset from the freshwater Lake Stechlin with the objective to i) estimate the contribution of Chytridiomycota phytoplankton parasites and pollen-degrading saprotrophs to the total pelagic zoosporic fungal community, and ii) assess their diversity and seasonal dynamics in relation to host association and inferred lifestyle. By synergizing state-of-the-art methods with chytrid infection prevalence data, we provide new insights into chytrid diversity, ecology, and seasonal dynamics in a temperate lake.

## Materials and methods

### A schematic overview of the workflow is presented in figure S1

#### Lake sampling

The sampling period spanned 15 months from March 2015 to June 2016 in temperate, dimictic, and mesotrophic Lake Stechlin, Germany [31], [Supplementary Text S1, Fig. S1, Supplementary Table S1]. Two integrated water samples of the upper mixed water layer (6–14 m) were taken weekly or bi-weekly (except in August 2015 and February 2016) with a hose (5 cm diameter) or an integrating water sampler (HYDRO-BIOS IWS III, Kiel).

For environmental DNA extraction, volumes of 0.5–1 L of lake water were filtered onto 5 µm pore size polycarbonate filters (47 mm diameter, Merck Millipore, Germany) to enrich particle-associated fungi. All filters were stored in cryotubes at −80 °C until further processing. One integrated water sample (6 L) was gently concentrated in situ by a 25 µm-plankton net underwater and subsequently pre-filtered over a 280 µm sized mesh to remove mesozooplankton. A subsample of 50 mL served to screen for chytrid infections on phytoplankton, subsequent cultivation, and single-cell isolation. The rest (50 mL) was fixed with alkaline Lugol´s solution and stored at 4 °C for quantifying the the percentage of a host population infected by chytrids [32]. Chytrid sporangia were visualized using a dual staining protocol with Calcofluor White (CFW) and Wheat Germ Agglutinin, conjugated to Alexa Fluor 488 (WGA) [33]. Whenever possible, 300 individuals of each phytoplankton species with visible chytrid infection were counted by using an inverted microscope (Nikon eclipse Ti2, 400X, fluorescence channels CFW: 387/11 nm excitation and 442/46 nm emission, WGA: 482/35 nm excitation and 536/40 nm emission). In cases of low phytoplankton abundance, the whole Utermöhl counting chamber was screened. Samples for phytoplankton biomass quantification were collected separately as part of a routine monitoring program with bi-weekly or monthly intervals (Supplementary Text S2, Supplementary Table S2).

#### Single-cell isolation and cultivation of phytoplankton and pollen-associated chytrids

Individual, infected phytoplankton cells and pollen grains were picked using a 0.5–10 µm micropipette under an inverted light microscope (Nikon Eclipse TS100, 100X). Picked single cells were transferred and washed thrice in 0.2 µm filtered MilliQ water before being transferred into 0.5 mL PCR tubes (total volume: 1 µL of 0.2 µm filtered MilliQ water) and stored at −20 °C until further processing.

For establishing chytrid cultures, a similar procedure was used, where after washing, single phytoplankton cells with attached sporangia were transferred into wells of a 24-well plate containing each 1 mL of CHU-10 medium of a phytoplankton host culture or pollen suspension. After successful infection, phytoplankton-chytrid co-cultures were established and maintained as previously described [34]. Saprotrophic chytrids isolated from pollen were transferred and maintained in liquid mPmTG medium [35] (for cultivation details see Supplementary Text S3).

##### DNA extraction and sequencing

DNA of single infected cells was extracted using the Hot-SHOT extraction method [30] or Illustra Single Cell GenomiPhi DNA amplification kit (GE-Healthcare). DNA of culture isolates was extracted from zoospores (separated from host cells by filtration through a 10 µm nylon mesh) or from host chytrid co-cultures using the peqGOLD Tissue DNA Mini Kit (Peqlab Biotechnology GmbH, Germany) or Hot-SHOT extraction method [36]. The 28S and 18S rRNA genes of chytrids were amplified with primers LROR-LR5 [37, 38] and NS1-NS4 [39] or EF4-EF3 [40], using MyTaq Red DNA Polymerase as previously described in [29], and sequenced by Macrogen Europe. Sequences were quality-controlled and assembled using BioEdit [41]. Additionally, the rRNA operon of single cells was sequenced using Oxford Nanopore sequencing with primer pair NS1short and RCA95m, as described in [42] and 18 S rRNA and 28 S rRNA genes of single-cell Dolichospermum-MDA2-akinete were retrieved from shotgun metagenome sequencing (Supplementary Text S4, Willis et al. in revision). DNA extraction and sequencing methods for each isolate/single cell are listed in Supplementary Table S3.

### Phylogenetic analysis of culture and single-cell isolates

For phylogenetic analysis, we created datasets of 18S and 28S rRNA gene sequences containing environmental sequences of uncultured chytrids related to culture and isolate sequences. *Salpingoeca infusionum* and *Monosiga brevicollis* (Choanozoa) and *Nuclearia simplex* (Cristidiscoidea) were selected as outgroup taxa. Sequences were automatically aligned with MAFFT v. 7.475 [43], independently for each gene region. Ambiguously aligned regions were excluded using trimAl v. 1.2 [44] with a gappyout model. A concatenated alignment was generated and partitioned by genes for analysis with maximum likelihood (ML) methods. The ML tree was inferred using RAxML v. 8.2.12 [45] on Cipres Science Gateway [46]. For further details see Supplementary Table S8 and Supplementary text S5.

### Evaluation of chytrid host range and consumer strategy

To examine the host range of parasitic chytrid strains, cross-infection assays were performed as described in [29]. Briefly, 0.5 mL of zoospore suspensions (after filtration of a 7 days old, infected culture through a 10 µm plankton mesh) were added to 1 mL of exponentially growing phytoplankton host. The original chytrid host strain served as a reference. Each assay was performed in triplicates using 24-well plates. Visual inspection of the infection was performed by inverted light microscopy (Nikon Eclipse TS100). Cross-infection results of the following chytrid cultures have been published in previous studies: isolates STAU-CHY3 [34], SVdW-EUD1, SVdW-EUD2, SVdW-EUD3 [29], and SVdW-SYN-CHY1 [47]. In this study, two additional diatom parasite strains, Fragilaria-CHY1 and AST-CHY1, were evaluated for their infection potential on eight different host species, including five diatoms (*Fragilaria crotonensis, Ulnaria* sp. (former *Synedra* sp. [47]), *Asterionella formosa, Aulacoseira ambigua*, *Aulacoseira granulata*) and three green algae (*Yamagishiella unicocca*, *Eudorina elegans, Staurastrum* sp.). Chytrid isolates Staurastrum-CHY4 and Staurastrum-CHY5 were tested on three desmid species (*Staurastrum* sp., *Closterium* sp., *Cosmarium* sp.). All parasitic strains were tested for their saprotrophic growth capability on pine pollen grains and artificial mPmTG medium [35]. All host-chytrid associations identified from single-cell data and cross infection assays were represented in an association matrix using the vegan package in R (Fig. 3). Based on the cross-infection results we categorized a chytrid species as 1) “specialist parasite” when it infected solely a single phytoplankton species, 2) “generalist parasite” when it was associated with more than one phytoplankton species and 3) “facultative parasite” when it was found in association with both phytoplankton and pollen, and/or was capable of growth on mPmTG medium or senescent phytoplankton.

### Field experiment: in situ pollen baiting

Pollen was collected on a dry and canopied surface close to Lake Stechlin in spring 2015. Most pollen were from *Pinus sylvestris*, but also birch and beech trees. A mixed pollen solution was prepared by adding 200 mg pollen in 750 mL sterile MilliQ water (0.27 g L^−1^). Thirty-five mL of this solution was transferred to custom-made baiting chambers and incubated just below the surface in Lake Stechlin for 1 week (15th to 22nd May 2015) at 4 locations: 1) littoral zone macrophyte area, 2) littoral zone reed stand, 3) littoral zone above sandy sediment, and 4) pelagic zone. Three replicates were deployed at each littoral and six at the pelagic site, yielding 15 samples in total. After incubation, pollen was rinsed to remove non-attached organisms and re-suspended in 40 mL of 0.2 µm filtered lake water. Twenty mL of pollen solution were filtered onto 5 µm pore size polycarbonate filters (47 mm diameter, Merck Millipore), plunged into liquid nitrogen, and stored at −80 °C until further processing. More details on the set-up and handling are given in Supplementary Text S6, Fig. S2).

### DNA extraction and sequence data analysis of lake and in situ pollen baiting samples

Genomic DNA was extracted using a CTAB-phenol-chloroform-isoamyl alcohol/bead-beating protocol (modified after [48], Supplementary Text S7). PCR, library preparation, and sequencing were performed by LGC Genomics (Berlin, Germany). Briefly, the D1 region of the LSU was amplified using forward primer ITS4ngsF (5’-GCATATCAATAAGCGSAGGA-3’) and reverse primer LF402R (5’-TTCCCTTTYARCAATTTCAC-3’) (modified after [49]), followed by library preparation and sequencing (2 × 300 bp) on a MiSeq (Illumina) platform. A total of 42 lake samples and 15 pollen-bait samples were sequenced. Demultiplexed raw sequence data was quality checked and analyzed using the DADA2 package [50] in R using default parameters (maxN = 0, maxEE = 2, truncQ = 2), generating sequences of about 350 nt.

To analyze the fraction of zoosporic fungal diversity identified using the targeted cultivation-dependent and -independent approaches, all generated field ASVs (amplicon sequence variants) were compared against all sequences obtained from culture strains, single cells, and pollen-baiting experiment. Additionally, sequences and ASVs were compared to the NCBI nt database release 246: October 15 2021. A Lake Stechlin ASV was considered identical to a sequence generated in this study or from the NCBI nt database when reaching a sequence similarity of ≥99% and a minimum sequence coverage of 85%.

ASVs from in situ pollen baiting were taxonomically assigned by manually searching the NCBI nt database using BLAST (BLAST + v2.10.0) (Supplementary Table S4). Initial taxonomic assignment of Lake Stechlin ASVs was done using the SILVA Online classifier with the LSU database v138 [51] (Supplementary Table S5). Fungal assignment followed the criteria given by [49] for the LSU D1 barcode. When sequence similarity of fungal ASVs assigned to one of the zoosporic fungal lineages Chytridiomycota, Blastocladiomycota, Aphelidiomycota and Rozellomycota was lower than 85% to a reference sequence, the ASV was manually verified by searching the NCBI nt database using BLASTn. Only ASVs with an 80% sequence similarity and 85% query coverage of a zoosporic fungal sequence in the NCBI nt database were treated as “zoosporic fungi”. Final taxonomic verification and sequence affiliation of zoosporic fungal ASVs was based on a phylogenetic approach (Supplementary Text S8, Figure S9). The extracted ASV abundance matrix of zoosporic fungi (including three unclassified ASVs that matched with the sequences from *Dolichospermum* spp. attached chytrids obtained in this study) was imported into R for further analysis (Supplementary Table S6). All sequence reads are available in the NCBI Sequence Read Archive (SRA) under BioProject PRJNA682007. Sequences from strains and single cell isolates were deposited under accession no. OL869010-OL869016; OM859415-OM859422 (18S Sanger), OL868971-OL869009 (28S Sanger), OL869133 (28S shotgun metagenome), OL869110 (18S shotgun metagenome), OL869111-OL869121 (Nanopore).

### Statistical analysis

Statistical analyses have been carried out using PASTv3.25 [52], unless stated otherwise.

Alpha diversity measures and principal coordinates analysis (PCoA) were calculated on a subsampled dataset including ASVs belonging to zoosporic fungi. All environmental samples were subsampled to 1000 sequences because of the high variation in sequencing depth. Subsampling was done using the ‘rrarefy’ function in the vegan package v2.5–7 [53] in R 3.6 [54], and samples with fewer reads were removed. The rarefied ASV table was Hellinger-transformed, and Bray-Curtis dissimilarities were used for PCoA analysis. Samples were sorted into seasons according to the meteorological calendar. Differences between seasons were analyzed with ANOVA for normally distributed data (Kruskal-Wallis when non-normally distributed) for alpha diversity and PERMANOVA [55] for beta diversity. The correlation between chytrid ASV47 (parasite on diatom *Fragilaria*) and putative hyperparasite Rozellomycota ASV141 (Fig. S8) was determined by calculating Pearson correlation coefficient using R.

## Results

### Diversity of cultured isolates and single cells/colonies

**Fig. 1:**
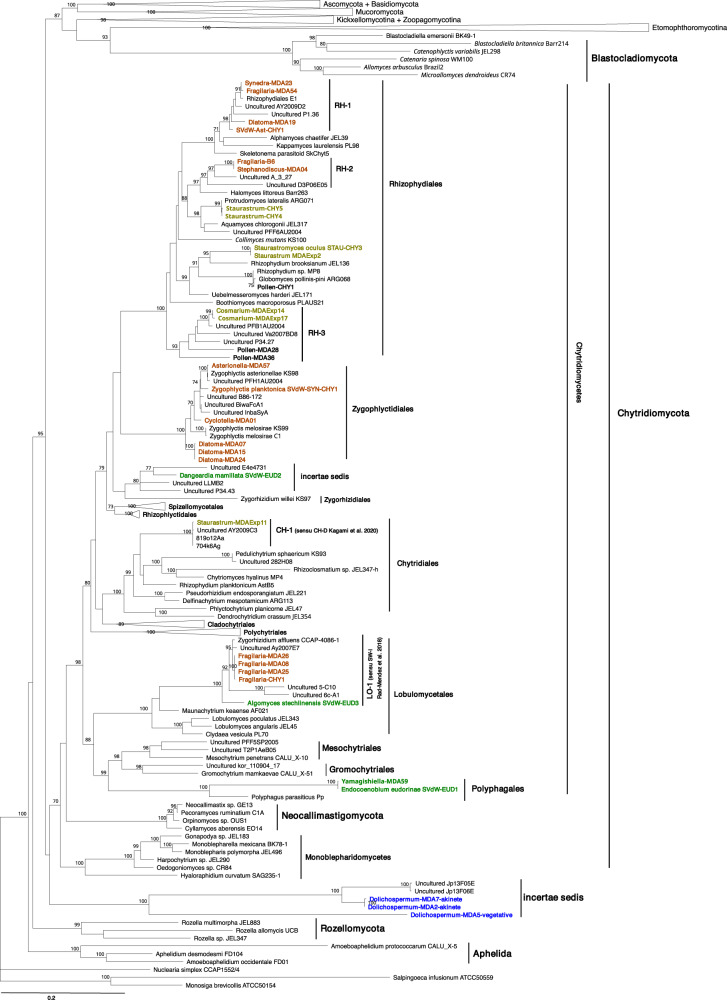
Maximum-likelihood tree of Fungi using concatenated rRNA gene sequences (18S, 28S). The maximum likelihood bootstrap values of 1000 repetitions are indicated at the nodes. Isolates and single cell sequences from this study are marked in bold and color coded according to their host/substrate; brown (diatom host), dark green (chlorophyte host), light green (desmid host), blue (cyanobacteria host), black (pollen substrate).

In total, 18 chytrid strains were isolated and 157 single-infected (host-chytrid) cells/colonies were collected between 2015–2017. Good quality sequences from single cells were obtained from 31 samples. This resulted in a reference library of 22 unique partial LSU sequences, of which 19 were associated with 14 phytoplankton host species and 3 with pollen (Table 1). All sequences obtained by cultivation or single-cell isolation belonged to the phylum Chytridiomycota, except two zoosporic *incertae sedis* fungi, which were associated with akinetes and vegetative cells of *Dolichospermum* spp. cyanobacteria (Fig. 1).

**Table 1:** Annotated chytrid reference sequences originating from cultivation and single-cell isolation, obtained from Lake Stechlin.

ID	Chytrid ID	Isolation date	Original host species isolated	Chytrid morphology	Chytrid phylogeny	Accession no. 18 S/28 S/nanopore (18S-ITS-28S)
1	Cyclotella-MDA01	6 Apr 2016	*Cyclotella* sp.	not determinable	*Zygophlyctidales* sp.1	OL869011/OL868972/OL869112
2	Asterionella-MDA57	22 Jun 2016	*Asterionella formosa*	*Zygophlyctis asterionellae*^*1*^	Zygophlyctidales; *Zygophlyctis asterionellae*^1^	OM859421/-/OL869111
3	Diatoma-MDA07 Diatoma-MDA15 Diatoma-MDA24 Diatoma-MDA30	20 Apr 2016 27 Apr 2016 4 May 2016 11 May 2016	*Diatoma tenuis*	not determinable	Zygophlyctidales; *Zygophlyctidales* sp.2	OL869016/OL868990/OL869117 OM859415/OL868991/- OM859416/OL868992/- OM859417/-/-
4	Stephanodiscus-MDA04 Stephanodiscus-MDA05 Fragilaria-B6 Synedra-A1	30 Mar 2016 30 Mar 2016 13 Apr 2016 13 Apr 2016	*Stephanodiscus* sp.	*Podochytrium cornutum*^*2*^	*Rhizophydiales* sp.1	-/OL868985/OL869115 -/OL868986/- OL869014/OL868983/- -/OL868984/-
5	STAU-CHY3^3^ Staurastrum-MDAExp2 Staurastrum-CHYA2 Staurastrum-CHYB1 Staurastrum-CHYC1	25 Jul 2015 28 Jun 2016 28 Jun 2016 28 Jun 2016 28 Jun 2016	*Staurastrum* sp.	*Staurastromyces oculus*	Rhizophydiales; *Staurastromyces oculus*	KY350147/KY350145/- OM859418/OL868999/OL869119 -/KY555729/- -/OL868997/- -/OL868998/-
6	AST-CHY1	2 Dec 2016	*Asterionella formosa*	distinct	*Rhizophydiales* sp.2	OL869010/OL868971/-
7	Synedra-MDA20 Synedra-MDA23	4 May 2016 4 May 2016	*Ulnaria* sp. (former *Synedra* sp.)	*Rhizophydium fragilariae*^4^	*Rhizophydiales* sp.3a	-/OL868988/- OL869015/OL868987/OL869116
8	Fragilaria-MDA54 (LSU 2 bp difference)	26 May 2016	*Fragilaria crotonensis*	*Rhizophydium fragilariae*^4^	*Rhizophydiales* sp.3b	-/OL868989/OL869121
9	Diatoma-MDA19	4 May 2016	*Diatoma tenuis*	cannot be determined	*Rhizophydiales* sp.4	-/OL868993/-
10	Staurastrum-Chy4 Staurastrum-Chy5	15 Sept 2016 15 Sept 2016	*Closterium* sp. *Cosmarium* sp.	*Protrudomyces lateralis*^*5*^	Rhizophydiales; *Protrudomyces lateralis*^*5*^	-/OL869003/- -/OL869004/-
11	Cosmarium-MDAExp14	28 Jun 2016	*Cosmarium* sp.	cannot be determined cannot be determined	*Rhizophydiales* sp.5a	OM859419/OL869001/OL869120
12	Cosmarium-MDAExp17 (LSU 3 bp difference)	28 Jun 2016	*Cosmarium* sp.		*Rhizophydiales* sp.5b	-/OL869002/-
13	SVdW-EUD2^6^	15 Jul 2015	*Yamagishiella unicocca*	*Dangeardia mamillata*	Order incertae sedis; *Dangeardia mamillata*	MG605054/MG605051/-
14	Staurastrum-MDAExp11	28 Jun 2016	*Staurastrum* sp.	cannot be determined	*Chytridiales* sp.1	OM859422/OL869000/-
15	FRA-CHY1 Fragilaria-A1 Fragilaria-A3 Fragilaria-B1 Fragilaria-B7 Fragilaria-MDA06 Fragilaria-MDA08 Fragilaria-MDA25 Fragilaria-MDA26 Fragilaria-MDA39 Fragilaria-MDA42	5 Mar 2015 27 Apr 2016 27 Apr 2016 20 Apr 2016 6 Apr 2016 13 Apr 2016 20 Apr 2016 4 May 2016 4 May 2016 11 May 2016 11 May 2016	*Fragilaria crotonensis*	*Chytridium versatile*/ “Species 3”^7^	*Lobulomycetales* sp.1	OL869012/OL868973/- -/OL868975/- -/OL868977/- -/OL868974/- -/OL868976/- -/OL868978/- -/-/OL869114 -/OL868981/OL869113 OL869013/OL868982/- -/OL868979/- -/OL868980/-
16	SVdW-EUD3^6^	2 Dec 2015	*Eudorina elegans*	*Algomyces stechlinensis*	Lobulomycetales; *Algomyces stechlinensis*	MG605055/MG605052/-
17	SVdW-EUD1^6^ Yamagishiella-MDA59 Yamagishiella-MDAExp1 Yamagishiella-MDAExp5 Yamagishiella-MDAExp6	9 Jun 2015 22 Jun 2016 28 Jun 2016 28 Jun 2016 28 Jun 2016	*Yamagishiella unicocca*	*Endocoenobium eudorinae*	Polyphagales; *Endocoenobium eudorinae*	MG605053/MG605050/- -/-/OL869118 -/OL868994/- -/OL868995/- -/OL868996/-
18	Dolichospermum-MDA2-akinet Dolichospermum-MDA7-akinet	9 Aug 2017 9 Aug 2017	*Dolichospermum* sp.	*Rhizosiphon akinetum*^8^	Fungi Incertae sedis sp.1	OL869110/OL869133/- -/OL869005/-
19	Dolichospermum-MDA5-vegetative	9 Aug 2017	*Dolichospermum* sp.	*Rhizosiphon crassum*^4^	Fungi Incertae sedis sp.2	-/OL869006/-
20	Pollen-CHY1	22 May 2015	pollen pinus	*Globomyces pollinis-pini*^9^	Rhizophydiales; *Globomyces pollinis-pini*^9^	OM859420/OL869009/-
21	Pollen-MDA28	11 May 2016	pollen other	cannot be determined	*Rhizophydiales* sp.6	-/OL869007/-
22	Pollen-MDA36	11 May 2016	pollen other	cannot be determined	*Rhizophydiales* sp.7	-/OL869008/-

References: 1 Seto et al. [47], ^2^Canter 1970, ^3^Van den Wyngaert et al. [34], ^4^Canter 1953, ^5^Letcher et al. [56], ^6^Van den Wyngaert et al. [29], ^7^Canter and Lund 1953, ^8^Canter [58]

Chytrid strains represented five species that have been identified or newly described as *Staurastromyces oculus* (Rhizophydiales) [34], *Endocoenobium eudorinae* (Polyphagales), *Dangardia mamillata* (incertae sedis), *Algomyces stechlinensis* (Lobulomycetales) [29], *Zygophlyctis planktonica* (Zygophlyctidales) [47]. Strains Staurastrum-CHY4 (Rhizophydiales) and Pollen-CHY1 (Rhizophydiales), were identified as known species *Protrudomyces lateralis* and *Globomyces pollinis-pini*, respectively [56]. The remaining strains represent yet undescribed taxa. Strain Fragilaria-CHY1 (Lobulomycetales), parasitic on the diatom *Fragilaria crotonensis*, together with single-cell sequences retrieved from the diatom *Fragilaria* showed a close affiliation to *Zygorhizidium affluens*, a known parasite of the diatom *Asterionella formosa* [57]. Strain AST-CHY1, parasitic on *Asterionella formosa*, was placed within the novel clade RH-1 together with single-cell sequences from other diatom parasites, related to Alphamycetaceae and Kappamycetaceae (Fig. 1). Strain Fragilaria-B6 was isolated from a single-infected diatom cell belonging to *Stephanodiscus*, but could be maintained in the lab on senescent *Fragilaria* and *Ulnaria* diatoms. Its partial LSU sequence was identical to that of the single-cell sample Stephanodiscus-MDA04, forming a novel clade together with two uncultured clones from oxygen-depleted marine sediment and paddy field soil (RH-2), related to Halomycetaceae, within the Rhizophydiales. We identified another novel clade RH-3 within Rhizophydiales including single-cell sequences of desmid parasites and saprotrophs on pollen. Single cell isolate Staurastrum-MDAExp11 fell in the clade CH-D *sensu* Kagami et al. (2020) [28]. Parasites of diatoms *Cyclotella* and *Diatoma* represented new species within the order Zygophlyctidales (Fig. 1).

#### Host/substrate specificity

**Fig. 2:**
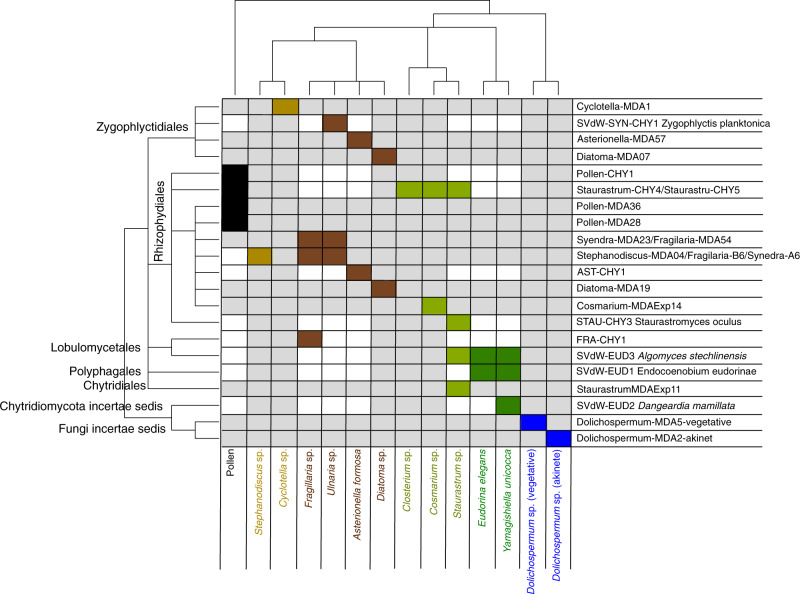
Host-chytrid association matrix based on experimental cross infection data and occurrence data. On the x-axis substrate (pollen) and phytoplankton host species and on the y-axis chytrid strains and single cell isolates, clustered according to their taxonomic relatedness. Rectangles indicate compatible host/substrate-chytrid pairs with the color code referring to host/substrate taxa; dark brown (pennate diatoms), light brown (centric diatoms), dark green (Chlorophyta), light green (desmids), blue (cyanobacteria), black (pollen).

Five strains were classified as specialist parasites (i.e., infecting only one host), two strains as generalist parasites, and three strains as facultative parasites (Fig. 2). The generalist parasite *Algomyces stechlinensis* had the most extensive host range, including two members of Chlorophyta and one desmid. The desmid *Staurastrum* sp. displayed the highest diversity of associated chytrids (four species).

### Fungal community associated with pollen: pollen-baits

We detected 51 fungal ASVs in the in situ pollen bait experiment, the majority were assigned to Chytridiomycota (75%), followed by Ascomycota (10%), Rozellomycota (6%), Blastocladiomycota (2%), and Mucoromycota (2%) (Supplementary Table S4). Within Chytridiomycota, members of Rhizophydiales were most abundant (95%). The ten most abundant ASVs represented 98% of the sequences, nine of which belonged to Rhizophydiales and one to Rozellomycota. The most abundant ASV matched with a single cell sequence of Pollen-MDA36. None of the top ten most abundant ASVs matched with any described facultative or saprotrophic chytrid species, but they were highly similar (96–99.7%) to sequences from other uncultivated pollen-associated chytrids **(**Supplementary Table S4**)**.

### Community composition of lake fungi

We determined 1741 ASVs in 42 pelagic samples of Lake Stechlin collected between March 2015 and June 2016. This period included two diatom spring blooms, and two “pollen rain” events. Among these ASVs, 1545 (89%) were classified within the fungal kingdom. The highest proportion of fungal ASVs belonged to Ascomycota (43%), followed by Basidiomycota (30%), Chytridiomycota (18%), Mucoromycota (3%), and Aphelidiomycota (1%). Rozellomycota, Neocallimastigomycota, and Blastocladiomycota made up together only 1% of the fungal community and 3% of fungal ASVs belonged to Fungi *incertae sedis* (Supplementary Table S5, Fig. S4).

### Illuminating the “dark matter” zoosporic fungi

We identified the host-substrate association of 26% (83 ASVs) of all zoosporic fungi in Lake Stechlin (319 ASVs), including Chytridiomycota, Blastocladiomycota, Aphelidiomycota, and Rozellomycota. Almost two-thirds of assignments were derived from our targeted approaches (cultivation/single cells 57%, in situ baiting 43%) and one-third stemmed from public reference databases. In total, 11.3% of ASVs were associated with pollen, and 13.2% of ASVs with phytoplankton (10.7% diatoms, 1.5% green algae, and 1% cyanobacteria). 1.5% of ASVs were associated with multiple substrates, i.e., green algae/pollen and green algae/diatoms (Fig. S5). When considering ASV sequence abundance instead of number, we could identify 68.5% of total zoosporic fungal reads. The majority of reads were associated with pollen (34%) and diatoms (30%), and only 1.1, 0.1, and 3.3% were associated with green algae, cyanobacteria, and multiple substrates, respectively (Fig. S5).

### Temporal dynamics of lake fungi abundance and prevalence of infection

**Fig. 3:**
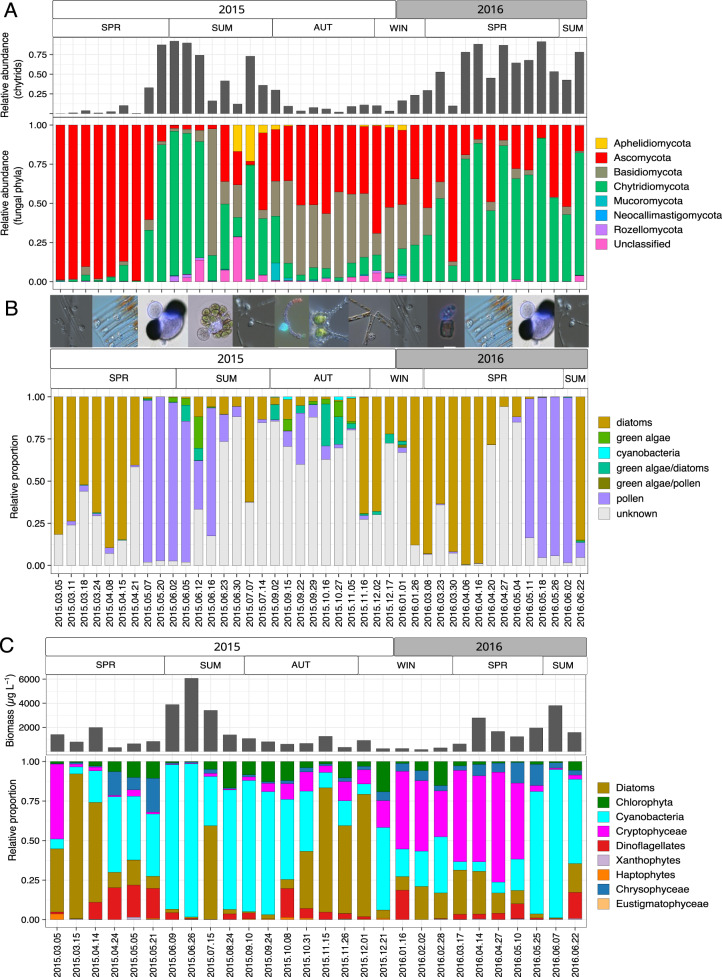
Seasonal dynamics of the fungal and phytoplankton community in Lake Stechlin. Fungal phyla and their relative abundance (**A**), identified zoosporic fungi substrate associations, including microscopy images illustrating the the succession of different phytoplankton/substrate-chytrid pairs (**B**) and biomass and relative proportions of phytoplankton taxa (**C**). Note different dates on X-axis for the lower phytoplankton plot.

In early spring 2015, the fungal community was dominated by Ascomycota associated with the diatom spring bloom, whereas Chytridiomycota dominated the fungal community during both “pollen rain” events in late spring 2015 and 2016, and during the diatom spring bloom in 2016. In summer, the fungal community was more diverse including the presence of Aphelidiomycota and a higher proportion of unclassified fungi. Autumn and winter periods were dominated by Ascomycota and Basidiomycota, with Chytridiomycota increasing in relative abundance towards January (Fig. 3A).

**Fig. 4:**
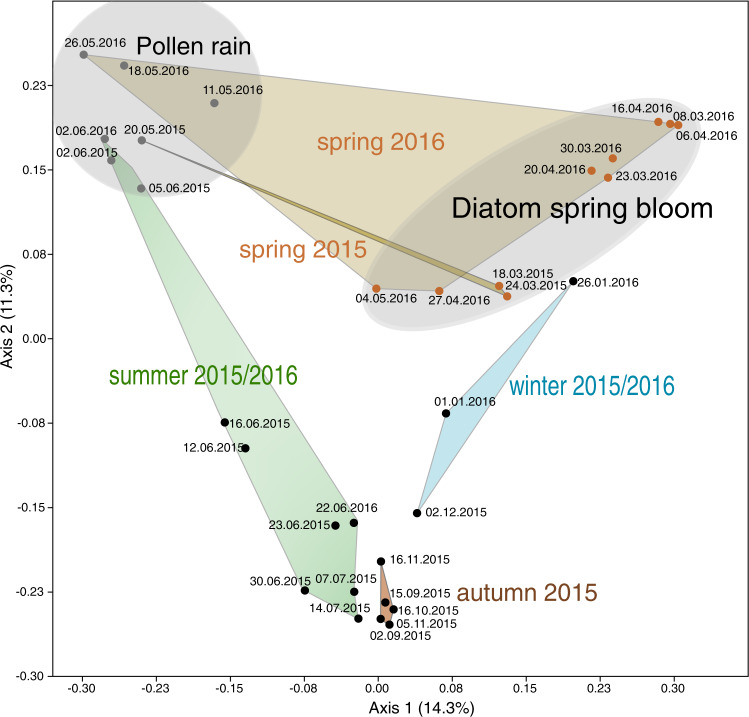
PCoA of zoosporic fungal composition over the sampling period across seasons (spring summer, autumn, winter) 2015 to 2016. Transparent gray areas indicate sampling dates during diatom spring bloom and pollen rain periods.

**Fig. 5:**
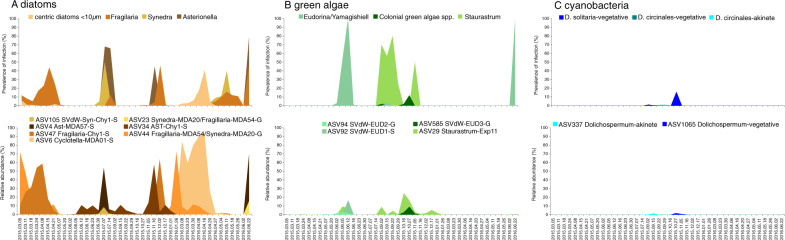
Temporal dynamics of the most abundant parasitic chytrid ASVs and prevalence of infection on the respective host species. Parasitic chytrids associated with diatoms (**A**), with green algae (**B**), and cyanobacteria (**C**).

The zoosporic fungal community in Lake Stechlin exhibited clear seasonal dynamics (Figs. 3B and 4, PERMANOVA Bray Curtis, *p* < 0.001). In spring, 72% ± 0.17 (2015) and 62% ± 0.40 (2016) of zoosporic fungal sequences matched diatom- or pollen-associated chytrids. During spring diatom blooms, parasitic chytrids dominated the community (Fig. 3B) and only a small proportion (<2%) was attributed to pollen-associated chytrids. Two parasites infecting the diatom *Fragilaria*, namely specialist Fragilaria-CHY1 (LO-1, ASV47) and generalist Fragilaria-MDA54/Synedra-MDA20 (RH-1, ASV23-ASV44), capable of infecting *Fragilaria* and *Ulnaria*, equally dominated the zoosporic fungal community (17–82%) during the spring bloom in 2015 (Fig. 5A). During this time, *Fragilaria* represented 2–10% of the total phytoplankton biomass (Supplementary table S2), and prevalence of infection was 5–44% (Fig. 5A). In spring 2016, both parasites were present in much lower relative abundance (1–12%), and during this time, *Fragilaria* did not exceed 1% of the total phytoplankton biomass and prevalence of infection reached 16%. Instead, *Cyclotella* sp. (a small centric diatom) was highly impacted by chytrids (max. prevalence 41%) with ASV6 corresponding to single-cell Cyclotella-MDA01 (max. relative abundance 91%) dominating the fungal community (Fig. 5A). Diatom bloom decay and the onset of pollen rain were reflected by a shift towards saprotrophic pollen-degrading chytrids, reaching 97% (±0.09) and 92% (±0.07) of the total reads in May 2015 and 2016, respectively (Fig. 3B).

Summer and autumn samples showed the highest zoosporic fungal diversity being significantly higher compared to spring 2016 (summer 2016-spring 2016: *p* < 0.001; autumn 2016-spring 2016: *p* < 0.001, Supplementary data, Fig. S6). These seasons displayed a mix of saprotrophic, parasitic, and facultative parasitic chytrids associated with major phytoplankton groups (green algae, cyanobacteria, diatoms) and contained a higher percentage of “unknown” sequences (Fig. 3B). *Zygophlyctis asterionellae* (ASVs 4, 96, 98, 102, 125, 149, 220), a host-specific parasite on *Asterionella*, reached a high relative abundance in summer, coinciding with two chytrid epidemics on the diatom *Asterionella* and infecting up to 68% (2015) and 78% (2016) of the population (Fig. 5A). *Zygophlyctis planktonica* (ASVs 105, 255, 416, 630, 668), a closely related but host-specific chytrid for *Ulnaria sp*. [47], showed a similar temporal pattern as *Zygophlyctis asterionellae* (Fig. 5A). A second parasite of *Asterionella*, AST-CHY1 (ASVs 34, 1302), frequently occurred during the whole year, albeit in low relative abundance (0.2–11%), even when the *Asterionella* biomass was very low or non-detectable and no infected *Asterionella* cells were detected by microscopy (Fig. 5A). In autumn (16th November 2015), ASV 34 dominated the zoosporic fungal community (57%) when *Asterionella* was present at relatively low biomass compared to summer (Fig. S7), but 40% of the population was infected. Chytrids associated with green algae, e.g., *Dangardia mamillata* (ASV94) and *Endocoenobium eudorinae* (ASV92), were mainly present in summer and *Algomyces stechlinensis* (ASV585) in autumn. Patterns of prevalence of infection on the host species followed occurrence patterns of the respective parasites (Fig. 5B). *Staurastrum*-MDAExp11 (ASVs 29, 1003) was present on different occasions throughout the year. The highest relative abundance occurred during autumn when *Staurastrum* was present and infected, but did not match with the prevalence of infection pattern (Fig. 5B). Parasites of cyanobacteria, infecting *Dolichospermum solitaria* and *D. circinalis* vegetative cells and akinetes only occurred in autumn and at low relative abundance (<2%). Prevalence of infection on *D. solitaria* and *D. circinalis* ranged from 1–16% (note: the maximum value was only based on six filaments) (Fig. 5C). Microscopic observations confirmed the absence of chytrid infections on *Dolichospermum* spp. during summer blooms when total cyanobacteria biomass was highest (Fig. S7), but the relative proportion of *D. solitaria* in the *Dolichospermum* community (total biomass) was lower, i.e., max. 0.2% in summer vs. max. 24% in autumn (Supplementary table S2).

During winter, a mixed community of saprotrophic and parasitic chytrids persisted with higher proportions of diatom parasites (Fig. 3B). Prevalence of infection on diatom species was low (<2%) in winter compared to other seasons (Fig. 5A).

## Discussion

### Illuminating “dark matter” zoosporic fungi

Over the course of 15 months we identified zoosporic fungi that exhibited varying degrees of phytoplankton host specific parasitism, and saprotrophy on pollen. A high turnover of fungal diversity, driven by changes in autochthonous and allochthonous available carbon, is remarkable and has implications for both the diversity of fungi and associated phytoplankton. We show that chytrid epidemics on diatom species (including small edible species) occur throughout the year and are driven by multiple parasite species that either co-occur or occupy different temporal niches. Revealing those dynamics was only made possible by linking targeted isolation approaches, laboratory infection assays, microscopy, and metabarcoding which greatly improved our ability to assign ecological functions to environmental sequences.

Of all zoosporic fungal ASVs, 26% could be assigned to parasitic phytoplankton-infecting or saprotrophic pollen-degrading lifestyles. This assignment would be substantially lower (<10%) based on the current status of the NCBI sequence database. Moreover, this study obtained the first sequences of two parasitic chytrids tentatively identified as *Rhizosiphon akinetum* and *R. crassum* associated with the nuisance cyanobacterium *Dolichospermum* [12, 58] and revealed their putative phylum-level phylogenetic novelty. Phylogenomic analysis is necessary to clarify their precise phylogenetic position. We further identified novel diatom-specific parasites within Zygophlyctidales, Rhizophydiales, and Lobulomycetelaes, emphasizing the large potential of phytoplankton-associated fungal parasites to fill current research gaps concerning aquatic fungal diversity and taxonomy. Besides two sequences obtained from the pollen baits, the majority of pollen-associated fungal ASVs were not assigned to any known species. Although most reference sequences belong to saprotrophic chytrids, such low agreement reflects that many saprotrophic chytrids in the databases have been isolated primarily from soil, ponds, and wetlands [56, 59] and that lake ecosystems harbor unique, uncharacterized pollen-degrading chytrids. Moreover, pure pine or sweet gum pollen is commonly used for isolating saprophytic chytrids [59, 60], whereas our study used natural pollen bait originating mainly from pine trees, but also including pollen from other tree species (presumably birch and beech) collected from the local environment. Our result suggests that the diversity of pollen-degrading chytrids is likely to be underestimated when only baiting with single pollen types and that saprophytic pollen degrading chytrids display some degree of specificity for different pollen types. Importantly, ASVs assigned to either parasitic phytoplankton-chytrid and saprotrophic pollen-chytrid interactions made up almost 70% of all zoosporic fungal reads in Lake Stechlin, suggesting that they are major components of the zoosporic fungal community. We do point out that, in accordance with previous freshwater studies [61, 62], Ascomycota and Basidiomycota presented the majority of fungal ASVs (see Supplementary Text S9 for more details). The combination of targeted isolation with environmental sequencing, proven successful in our study system, could be applied to any type of ecosystem and fungal group. Transferability of such an approach for higher fungi will depend on identification and isolation expertise of researchers. The heterogeneous morphologies and often complex life cycles of Ascomycota and Basidiomycota, i.e., from small, free living single celled yeast to large substrate associated filamentous hyphae, may provide additional challenges compared to the rather simple life cycle and morphology of attached sporangial forms of chytrids.

### Specialist vs. generalist

Our cross-infection experiments showed that strain Fragilaria-CHY1 was host-specific for *Fragilaria*. Its partial SSU, LSU, and ITS sequences were, however, almost identical to the recently rediscovered and sequenced species *Zygorhizidium affluens*, parasitic on the diatom *Asterionella formosa* in Lake Pavin, France [57]. Nanopore sequencing of the rRNA operon from single cells confirmed partial SSU, LSU, and ITS sequences being identical to Fragilaria-CHY1, but also showed several introns in the SSU region. Host specificity of *Z. affluens* has not been investigated, however, differences between *Z. affluens* and Fragilaria-CHY1 in host specificity and introns may suggest genetic isolation with ongoing diversification and host specialization [63]. Or, as seen in other host-parasite systems, both specialist and generalist strains likely coexist [64]. In case Fragilaria-CHY1 would have a more generalist lifestyle, we would expect it to occur also during times when *Fragilaria* is absent or not infected, indicating its potential to reproduce on alternative host species. However, the corresponding prevalence of infection pattern on *Fragilaria* with the presence and relative abundance of ASV47 (Fragilaria-CHY1) and the absence of ASV47 during both epidemics on *Asterionella*, supports its host preference for *Fragilaria* (Fig. 5A). On the contrary, AST-CHY1 was only infective on *Asterionella* in our cross-infection assays, while the corresponding ASV34 frequently occurred even when *Asterionella* was absent or not infected (Fig. 5A), pointing to a generalist lifestyle for this parasite. It should be noted that our cross-infection assays used a combination of a single clonal chytrid strain with a single clonal host strain, thus, extrapolating to the population level advises caution. Another issue that needs consideration is phylogenetic resolution. The LSU D1 marker showed limitations to resolve the closely related diatom parasites within clade RH2 (ASV23 matched 100% with Syn-MDA20-Fra-MDA54 but had only a 2 bp mismatch with Ast-Chy1, whereas ASV34 matched 100% with Ast-Chy1 and had a 2 bp mismatch with Syn-MDA20-Fra-MDA54 and only 1 bp mismatch with Diatom-MDA19). Culture isolates of Syn-MDA20-Fra-MDA54 and Diatom-MDA19 are needed to resolve better inter- vs. intraspecific variability within this clade. Interestingly, Staurastrum-MDAExp11, associated with desmid *Staurastrum* sp. in Lake Stechlin had almost identical LSU (99.77%) sequences as single-cell chytrids (819o12Aa and 704k6Ag) associated with two species of the diatom genus *Aulacoseira* in Lake Inba (Japan) [28]. The discrepancy between the patterns of prevalence of infection on *Staurastrum* and the presence of Staurastrum-MDAExp11 (ASV 29) support its more generalist lifestyle (Fig. 5B). Whether this represents a rare case of a generalist chytrid with an inter-taxonomic host range would require additional isolation and cross infection assays.

### Seasonal dynamics of zoosporic fungi

The zoosporic fungal community in Lake Stechlin showed a clear seasonality with distinct winter-spring, summer, and autumn communities. Our observations support the hypothesis that saprotrophic chytrids are related to the input of allochthonous organic matter (i.e., pollen) and parasitic chytrids to the seasonal dynamics of their phytoplankton hosts [4, 12, 32]. Chytrid infection on phytoplankton occurred throughout all seasons and years examined. Whereas infected phytoplankton could not be observed by microscopy on June 23rd^,^ 2015, and January 26th^,^ 2016, metabarcoding revealed the presence of ASVs matching with diatom parasites that accounted for 10 to 80% of the zoosporic fungal community (Fig. 3B, Supplementary tables S7).

Different seasonal patterns were detected between multiple chytrid parasites sharing the same host. Whereas Rhizophydiales sp. (AST-CHY1) and *Zygophlyctis asterionellae* parasites of *Asterionella* dominated in different seasons, parasitic generalist (Fragilaria-MDA54/Synedra-MDA23) and specialist (Fragilaria-CHY1) of *Fragilaria* also coexisted, though specialists are expected to be superior competitors on a common diatom host [28]. Species-specific environmental optima may drive such different seasonal dominance patterns [65–67] and the presence of host-specific hyperparasites could provide another mechanism for the coexistence of multiple parasite species on the same host population [68, 69]. Microscopic observation identified a putative Rozellomycota hyperparasite encysted on a chytrid sporangia infecting *Fragilaria* (Supplementary Fig. S8). Additionally, a strong correlation (Pearson´s *R* = 0.97, *p* < 0.001) was found between specialist Fragilaria-CHY1 (ASV47) and the most abundant Rozellomycota ASV (ASV141), suggesting a putative Rozellomycota hyperparasite infecting chytrid Fragilaria-CHY1, as described previously [70].

Contrary to obligate parasites, ASVs matching with facultative parasites only occurred in a few samples and never reached high relative abundances, i.e., strain Fragilaria B6/single-cell Stephanodiscus-MDA04 (1 sample, 0.4%), Staurastrum-CHY4 (5 samples, max. 1.7%), *Aquamyces chlorogonii* (1 sample, 0.5%). Whereas obligate parasites are likely to be superior competitors in the upper pelagic zone associated with active phytoplankton growth, the importance of facultative parasites may increase with depth, i.e., with increasingly senescent or dead cells of sinking phytoplankton in the hypolimnion.

Chytrid epidemics have been mostly reported from large (e.g. inedible) bloom forming diatom species [12, 71, 72], but we observed that also small-sized diatoms (e.g. *Cyclotella* spp.) are highly impacted by chytrids. Single-cell Cyclotella-MDA01 constituted the third most abundant zoosporic fungal ASV considering the entire sampling period. During the 2016 spring bloom, this chytrid dominated the overall fungal community, highlighting the importance of chytridiomycosis also for smaller and thus potentially more edible diatoms. This is of great relevance as lake warming mainly favors small-sized planktonic diatom species, particularly within the genus *Cyclotella* [73]. Yet, the resulting effects for higher trophic levels, e.g. via the mycoloop [22] require further investigations.

In addition to parasites, we show that also saprotrophic chytrids affect the seasonal succession of plankton communities. Over two consecutive years, the transition from the spring diatom bloom to a clear water phase with massive pollen input was consistently reflected by a shift from parasitic- to saprotrophic-dominated chytrid communities. Pollen input often occurs during the clear-water phase when phytoplankton biomass and nutrient concentrations are low [74] and thus represents an important nutrient input in spring-summer in many temperate and boreal lakes [75]. For example, in Lake Stechlin, pollen rain accounts for nearly half of the yearly atmospheric phosphorus input [76]. Whereas pollen grains are hardly ingested by zooplankton, saprotrophic chytrids render this otherwise inaccessible food source available to grazers in the form of readily edible chytrid zoospores [77, 78]. Such a mycoloop [22] effectively channels allochthonous organic matter to higher trophic levels such as zooplankton, which in particular is important during the clear water phase when phytoplankton prey abundance is low.

Summer represented a transitional period leading to a more diverse saprotrophic and parasitic chytrid community which culminated in autumn when zoosporic fungal diversity was highest. Microscopy confirmed widespread chytrid infections on various phytoplankton taxa (highest number of infected species recorded in autumn; Supplementary Table S7). A similar pattern has been observed in other temperate lakes [6, 12] where a high phytoplankton diversity in autumn but at lower abundance compared to spring is suggested to favor the co-existence of a diverse community of host-specific parasites. However, due to low cell abundances, we were not able to capture this phenomenon with our isolation approach. Moreover, Aphelidiomycota, present in summer, are known parasites of green algae, yellow-green algae, and diatoms [79, 80] that may have possibly been overlooked in microscopic studies due to their intracellular infection stages. However, the relative abundance of Aphelidiomycota remained low (Fig. 5B and Fig. S4) indicating that chytrids represent the major zoosporic fungal phytoplankton parasites.

During winter, despite low phytoplankton biomass, parasitic chytrids on diatoms made up a substantial portion of the zoosporic fungal community. As water temperatures below 3°C may inhibit chytrid infection [65, 81], cold winters with ice coverage and no or little snow provide a disease-free window of opportunity for diatom growth [15]. In Lake Stechlin, the observed low levels of infected diatoms during winter reflect the importance of cold winters for diatom spring bloom development. Gradual loss of this environmental refuge, e.g. by increasingly warmer winters, may contribute to earlier and less intense diatom spring blooms and thus may lead to possible shifts in phytoplankton community composition during the following season [82–84]. As the bloom inoculum affects the subsequent phytoplankton dynamics in spring, the loss of the winter refuge from chytrids infection exemplifies the far-reaching consequences of gradual lake warming for plankton community dynamics, trophic interactions, and consequently ecosystem functioning [85].

### Summary

This study represents a unique effort to link zoosporic fungal sequence diversity and consumer-resource interactions in the mixed, pelagic zone of Lake Stechlin. We demonstrate a high turnover of zoosporic fungal diversity, driven by changes in autochthonous and allochthonous available carbon and provide evidence that phytoplankton-parasites and saprotrophic pollen degraders are key components of the zoosporic fungal community. Chytrid epidemics on diatoms (including small edible species) occur throughout the year and are driven by multiple parasite species that either co-occur or occupy different temporal niches. Revealing those dynamics was only made possible by linking targeted isolation approaches, laboratory infection assays, microscopy, and metabarcoding which greatly improved our ability to assign ecological functions to environmental sequences. We highlight that successful identification of the most abundant zoosporic fungal ASVs in Lake Stechlin was largely accomplished by single cell and culture isolate sequencing. As long read metabarcoding and (meta)genomics are improving rapidly by getting more cost-efficient, they will ultimately solve single marker choices for complex environmental samples, providing increased resolution and reduced taxonomic biases. Coupling these third-generation sequencing technologies to high quality reference sequences with rich metadata, as generated in this study, will enable a better exploration of spatial and temporal distribution of chytrids in temperate lakes worldwide.

## Supplementary information

Supplementary information

Supplementary figure S9

Supplementary table S1

Supplementary table S2

Supplementary table S3

Supplementary table S4

Supplementary table S5

Supplementary table S6

Supplementary table S7

Supplementary table S8

Dataset 2 zoosporic fungi ASVs

Datset 1 All ASVs

## Data Availability

Raw sequence data is available in the NCBI Sequence Read Archive (SRA) under BioProject PRJNA682007. Sequences from strains and single cell isolates have been deposited in GenBank under accession no. OL869010-OL869016; OM859415-OM859422 (18 S Sanger), OL868971-OL869009 (28 S Sanger), OL869133 (28 S shotgun metagenome), OL869110 (18 S shotgun metagenome), OL869111-OL869121 (Nanopore). All other data generated or analyzed during this study are included in this published article and its supplementary information files.
