# Supplementary material for: Seasonality of parasitic and saprotrophic zoosporic fungi: linking sequence data to ecological traits
Source: ISME J. 2022 Jun 28;16(9):2242–54. doi: 10.1038/s41396-022-01267-y (PMC9381765; doi:10.1038/s41396-022-01267-y)
Supplement: Supplementary file 1 — Supplementary information [file 41396_2022_1267_MOESM1_ESM.pdf]

Supplementary information: Seasonality of parasitic and saprotrophic zoosporic fungi: linking sequence data to ecological traits.

Silke Van den Wyngaert, Lars Ganzert, Kensuke Seto, Keilor Rojas-Jimenez, Ramsy Agha, Stella A. Berger, Jason Woodhouse, Judit Padisak, Christian Wurzbacher, Maiko Kagami and Hans-Peter Grossart

Materials and Methods

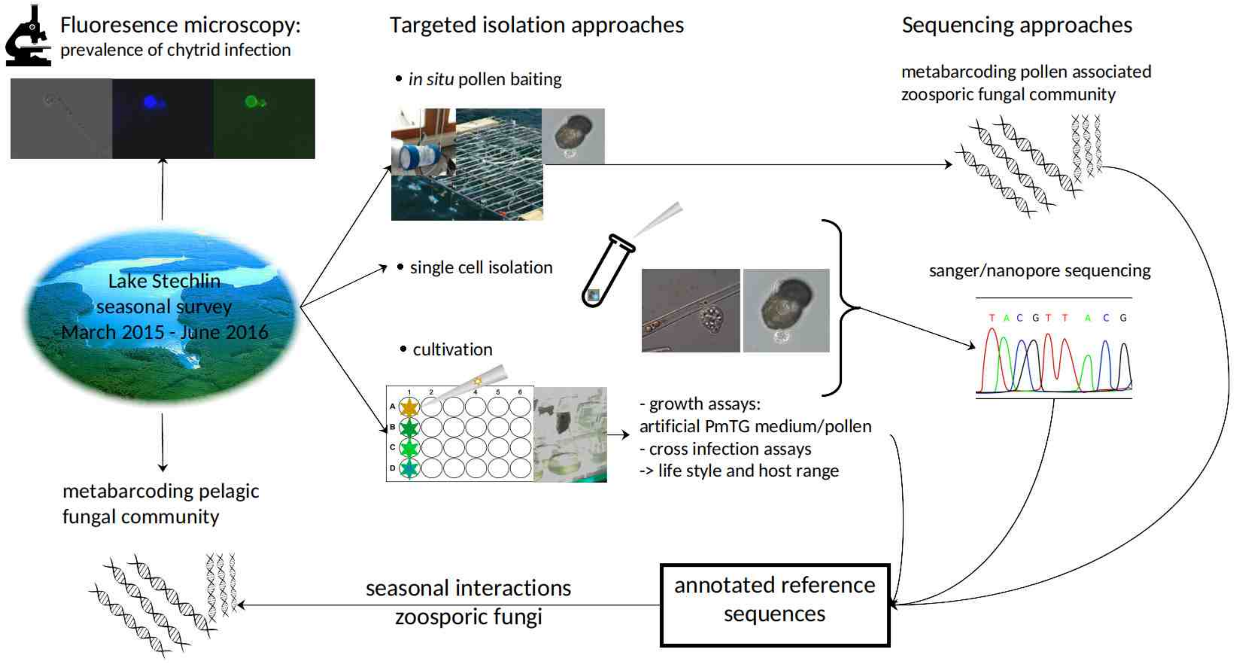

Figure S1 Schematic overview of the workflow and methods linking molecular and ecological approaches.

Text S1 Study site: Lake Stechlin

Lake Stechlin is a temperate, dimictic, and mesotrophic hard-water lake in North-East Germany (53°9′5.59″N, 13°1′34.22″E) with a surface area of 4.25 km^2 and a maximum depth of 69.5 m. The annual phytoplankton biomass of Lake Stechlin usually follows a bimodal pattern with a spring peak and a lower late summer peak [1] The spring bloom is typically dominated by centric and pennate diatoms, whereas the late summer assemblage is characterized by a heterogeneous mixture of phytoplankton taxa. The lake is situated in a forested area, and receives extensive pine pollen input in May and June (see image below). For lake Stechlin, a pollen input of ca. 2.7x10^4 kg year^{-1} has been estimated, contributing to an annual phosphorous load of 82.9 kg [2]

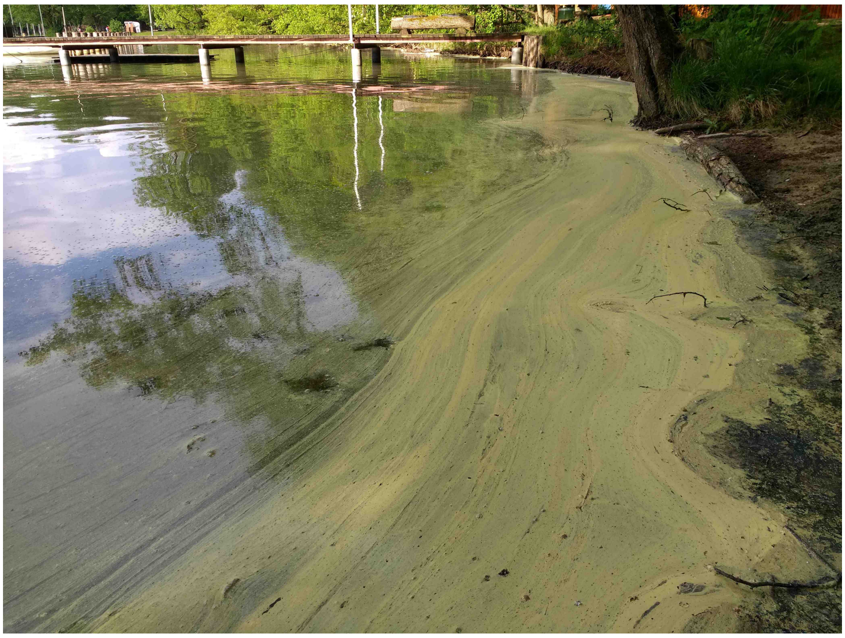

Fig. S2 Massive pollen accumulation in May at the shore of Lake Stechlin (northern Germany). Image taken 21.05.2019, image courtesy Silke Van den Wyngaert

Text S2 Phytoplankton monitoring Lake Stechlin

Phytoplankton samples were taken at the deepest site of the lake at 5 m increments between 0 and 25m depth (euphotic zone). The layered subsamples were pooled together and preserved in alkaline Lugol's solution before microscopic analysis. Phytoplankton species were identified using the most up-to date phycological manuals and literature. A minimum of 400 settling units (cells, filaments or colonies) were counted in an inverted microscope (Zeiss Axiovert 100, Oberkochen, Germany) in each Lugol-fixed sample giving a counting accuracy of + 10 % for total phytoplankton. Phytoplankton biomass was calculated based on cell volumes from the most similar geometric forms according to [3] using the Opticount cell counting software (available on internet https://science.do-mix.de/software_opticount.php)

Text S3 Culture conditions phytoplankton-chytrid co-cultures and saprophytic chytrids

Chytrid-host co-cultures were cultivated in 100 mL Erlenmeyer flasks containing 30-50 mL of the phytoplankton host culture. Both, host cultures and chytrid-host co-cultures were maintained in a CHU-10 medium in a climate-controlled room at a light intensity of ~40 µE s-1 m2 (cool-white fluorescent lamps) with a light:dark cycle of 16:8 h and temperatures between 17 to 19°C.

Saprophytic chytrid Pollen-CHY1 was cultivated in 100 mL Erlenmeyer flask containing 30-50 mL

mPmTG medium with antibiotics (Penicillin-Streptomycin) and kept on a shaker unit at ~20 degrees, non-controlled light conditions.

Recipe CHU-10 medium.

Supplement	Stock Solution* g L^{-1}	mL L^{-1}	µmol L^{-1} in the final medium
Na_2SiO_3 ($5H_2O$)	5.8	10	273
$Ca(NO_3)_2$ ($4H_2O$)	57.56	1	244
K_2HPO_4	10	1	57
$MgSO_4$ ($7H_2O$)	25	1	101
Na_2CO_3	20	1	189
add after autoclaving			
Fe-EDTA†	37	1	
F/2 Vitamins‡	see below	1	

*stock solutions stored at RT
†filtered through a 0.2 µm membrane filter (stored at 4°C)
‡sterile-filtered F/2 vitamins (stored at 4°C)

F/2 Vitamins

Supplement	Stock Solution mg mL^{-1}	/100ml dist. H_2O
Vitamin B12	5 mg 5 mL^{-1} distilled H2O	0.1 ml
Biotin	1 mg 10 mL^{-1} distilled H2O	1.0 ml
Thiamine HCl		20 mg

Recipe mPmTG medium

Peptonized milk	0.2g
Tryptone	0.2g
Glucose	1.0g
Distilled water	500 mL
Penicillin*	0.1g
Streptomycin*	0.1g

*add both antibiotics after autoclaving

Text S4 Shotgun sequencing and assembly of 18S rRNA and 28S rRNA genes of single-cell Dolichospermum-MDA2-akinete

Nextera DNA library was generated and sequenced using a 2x150bp Nextera Kit on the Illumina NextSeq instrument at the Genome Sequencing Facility, Walter and Eliza Hall Institute, Australia. Quality filtering (Q=15, length > 100 nt) removal of adapter and PhiX sequences were performed using the BBduk tool, available as part of the BBMap package (https://www.osti.gov/biblio/1241166-bbmap-fast-accurate-splice-aware-aligner). Filtered sequence reads were mapped against SILVA release 138.1 SSU and LSU databases using PhyloFlash v3.4 [4]. Aligned reads were reconstructed into partial ribosomal genes using SPAdes v3.13.1 as part of

the PhyloFlash pipeline.

Text S5 Phylogenetic analysis of culture and single-cell isolates

The ML tree was inferred using RAxML v. 8.2.12 [5] on Cipres Science Gateway [6]. We ran an analysis under the GTR + GAMMA model and used the "-fa" option to conduct a rapid bootstrap analysis with 1000 replicates combining 200 searches for the optimal tree.

The selection criteria for the sequences included in the phylogenetic tree were based on the following:

1) We included all major lineages (phyla, classes, and orders) of fungi.

2) At least 3~6 taxa were included in each lineage, except for minor groups such as Polyphagales, Mesochytriales, etc.

3) For the orders including our sequence data, we included as many as families and *incertae sedis* clades in the order (e.g. Chytridiales includes Chytridiaceae, Chytriomycetaceae, and family *incertae sedis* clade). But Rhizophydiales is partially insufficient because 18S rRNA gene sequences are lacking in some lineages.

4) For environmental sequences, (if existing) 2~3 environmental sequences related to our single cell or culture sequences were added (we looked for these sequences by BLASTn search).

For novel clade assignment, we follow the definition given by Tedersoo et al. (2017) [7] which defines a novel clade based on statistically supported branches (BS >70) featuring no described species and refers to these as clades following the International Code of Phylogenetic Nomenclature.

The tree was visualized with FigTree v1.4.4 (http://tree.bio.ed.ac.uk/software/figtree/) and edited in Inkscape 1.1.

Text S6 Pollen baiting experiment

Custom-made baiting chambers were constructed from 50 mL falcon tubes that were cut in half. The lids of the falcon tubes were stamped out with a metal puncher, creating a ring. A 10 µm nylon plankton mesh was used to cover both open ends of the falcon tube, and the lid rings were slid over the ends to keep the mesh in place. Steel weights were tied to the chambers with strings to keep

them submerged (see image below, top left). A mixed pollen solution was prepared by adding 200 mg pollen in 750 mL sterile MilliQ water (0.27 g L-1). The majority of pollen was from *Pinus sylvestris*, but also birch and beech trees. Thirty-five mL of this pollen solution were transferred to the baiting chambers. The chambers were then carefully filled up with 0.2 µm filtered lake water to avoid air bubbles upon closing and were incubated in Lake Stechlin for 1 week (15th to 22nd May 2015) at 4 habitats: 1) littoral zone macrophyte area, 2) littoral zone reed stand, 3) littoral zone above sandy sediment, and 4) pelagic zone. A platform frame was anchored in the pelagic zone at a distance of approximately 300m from the shore and ~20m depth. Baiting chambers were tied with string to the frame and submerged just below the surface water (~25cm). In the littoral areas, chambers were fixed along metal rails of a pier and submerged just below the surface water (~25cm) (see images below, top right and bottom).

Baiting chambers were collected from the lake in buckets filled with water from the same baiting locality and brought back into the laboratory where they were carefully opened to let the water inside the chamber flow out. The pollen remaining on the plankton mesh at the bottom of the chamber was rinsed to remove non-attached organisms and re-suspended in 40 mL of 0.2 µm filtered lake water. Twenty mL of pollen solution were filtered onto 5 µm pore size polycarbonate filters (47 mm diameter, Merck Millipore, Germany), plunged into liquid nitrogen, and stored at -80°C until further processing.

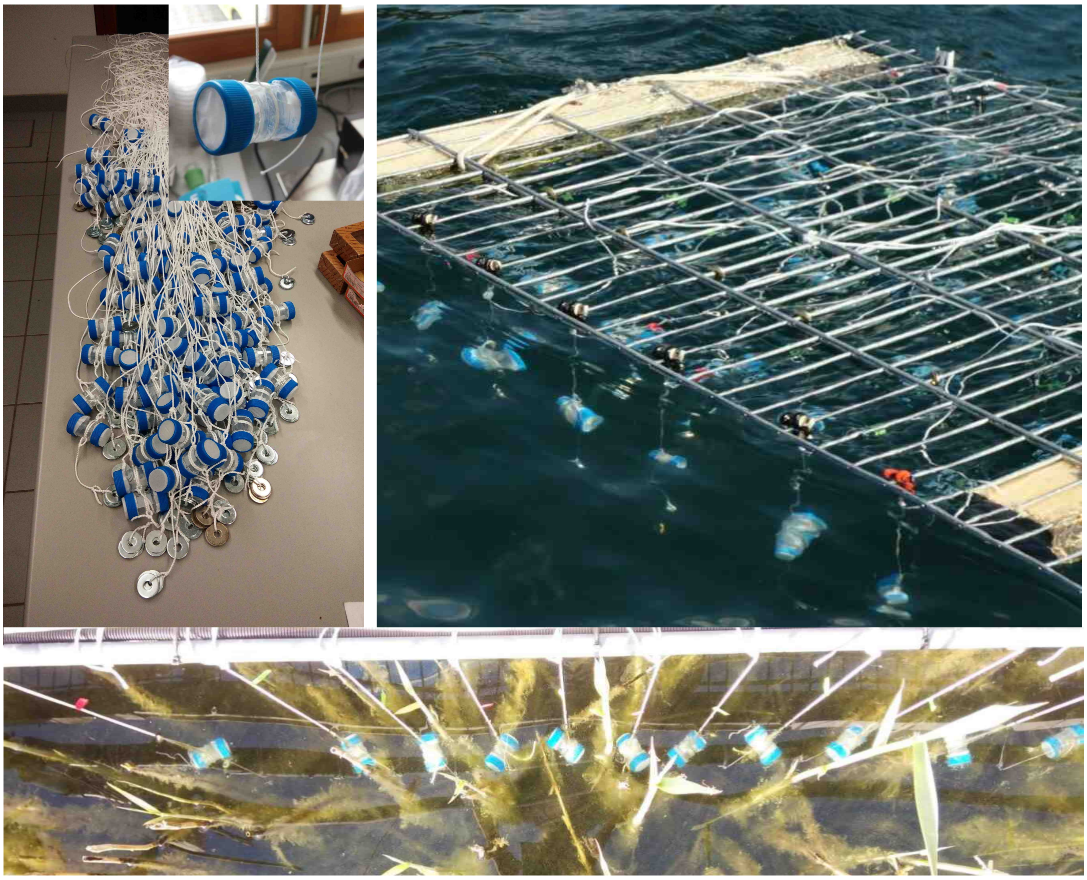

Fig. S3 Images of the custom made baiting chambers and their *in situ* deployment in the lake at pelagic and littoral sites in Lake Stechlin. Image courtesy Silke Van den Wyngaert

Text S7 DNA extraction of lake and pollen samples

Filters were homogenized in a bead beater using glass/zirconia beads (0.1, 0.7 and 3 mm), plus 600 µL extraction buffer (10% CTAB in 1.6 M NaCl mixed 1:1 with 240 mM K_2HPO_4/KH_2PO_4 buffer), 600 µL phenol:chloroform:isoamylalcohol (25:24:1) and 60 µL each 10% sodium dodecyl sulfate (SDS) and 10% N-lauroyl sarcosine. The aqueous phase was mixed with an equal volume of chloroform-isoamylalcohol (24:1) to remove any residual phenol. DNA was precipitated with 30% PEG 6000 in 1.6 M NaCl and the addition of 1 µL LPA (Sigma), followed by a centrifugation step. The resulting pellet was washed with 1 mL ice-cold ethanol (70%), dried briefly at 37 °C, resuspended in 50 µL DEPC-treated water, and stored at -80 °C until further processing.

Text S8 Taxonomic assignment of zoosporic fungal ASVs

Following the criteria outlined in (ref) for the LSU D1 barcode, a sequence similarity above 80% was considered sufficient for fungal assignment and a similarity above 85% for class-level assignment. After this step, all fungal ASVs were taxonomically assigned to one of the zoosporic fungal lineages Chytridiomycota, Blastocladiomycota, Aphelidiomycota and Rozellomycota. When fungal ASV sequence similarity was lower than 85% to a reference sequence, the ASV was manually verified by searching the NCBI nt database using BLAST. Only ASVs with a 80% sequence similarity and 85% query coverage of a zoosporic fungal sequence in the NCBI nt database were treated as "zoosporic fungi". Final taxonomic verification and sequence affiliation of the selected "zoosporic fungal ASVs" was based on a phylogenetic approach. Herefore, we created a 28S rRNA gene dataset representing taxa of chytrids and other fungal lineages. *Salpingoeca infusionum, Monosiga brevicollis* (Choanozoa), and *Nuclearia simplex* (Cristidiscoidea) were selected as outgroup taxa. Sequences were aligned with MAFFT v. 7.475 [8]. Ambiguously aligned regions were excluded using trimAl v. 1.2 [9] with a gappyout model. The ASV sequences were added into the alignment of represented taxa and aligned using "--add" and "—keeplenght" options of MAFFT v. 7.475. The maximum likelihood (ML) analysis was conducted using W-IQ-TREE web server [10]. We ran an analysis with the model "GTR+G4+F" and 1 000 replicates of ultrafast bootstrap [11] and Shimodaira-Hasegawa-approximate likelihood ratio (SH-aLRT) analysis. The tree was visualized with FigTree v1.4.4 (http://tree.bio.ed.ac.uk/software/figtree/), see Supplementary Figure S9_PhylogeneticTree_ASV where Lake ASVs are marked in blue and ASVs from *in situ* pollen baiting are marked in red.

Supplementary Results & Discussion

Text S9 Community composition of lake fungi

In accordance with previous studies, Ascomycota and Basidiomycota presented the majority of fungal ASVs (Figure S4) [12, 13] and in Lake Stechlin they mainly dominated the fungal community in autumn-winter. The majority of Ascomycota were affiliated to Pezizomycotina, an

ecologically diverse group of filamentous fungi that can have both terrestrial and aquatic members [14], whereas ASVs related to Ascomycota yeast (Saccharomycotina, Taphrinomycotina) were never abundant in the lake. The majority of Basidiomycota were affiliated to Agaricomycotina which include many decomposers of wood and leaf litter [15]. Many of these Basidiomycota species are dimorphic yeast species, e.g., their life cycle includes a yeast and a filamentous stage. Their relative increase in autumn is likely due to the introduction from wood-degrading Basidiomycetes from the surrounding forest, most of which release spores from July to November (pers. obs. of Wurzbacher et al. 2016).

Dominance of Ascomycota (Pezizomycotina, mainly from the order Hypocreales) was also observed during the early spring period in 2015. Hypocreales contains many species of aquatic hyphomycetes associated with leaf litter degradation. Some of these abundant Hypocreales ASVs, including the most abundant ASV1, only occurred in early spring 2015 whereas others (ASV7 and ASV9) also occurred during autumn. In the study of Taylor and Cunliffe (2016) [16] members of Hypocreales also revealed in some years, but not all, peak abundances in April in the surface water of the western English Channel. Impact of weather conditions and precipitation events may have caused an increase in the input of allochtonous organic matter in the pelagic and a potential shift towards higher relative abundances of Hypocreales species. Terrestrial filamentous fungi can be introduced into lakes through spores and pieces of mycelia during inflowing stream, rainwater and wind events [17]. Consequently, it is difficult to know if these fungi are truly aquatic [18]. However, rRNA analysis has shown that many of these fungi were also active in freshwater lakes [12]. The dominance of Chytridiomycota ASVs associated with phytoplankton blooms and pollen rains is consistent with the morphotype hypothesis, i.e. filamentous fungi are mainly associated with coarse particulate organic matter (CPOM) whereas zoosporic fungi become the dominant fungi in open water where fine particulate organic matter (FPOM) is the main type of organic material [19].

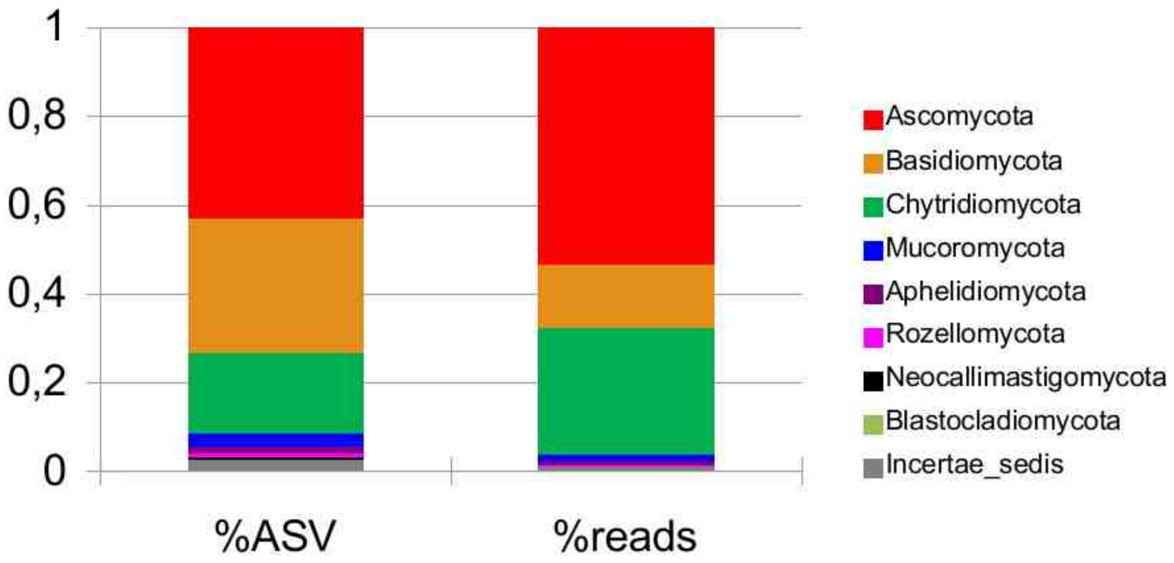

Fig. S4 Proportion of fungal phyla based on number of ASVs and number of reads in Lake Stechlin over the entire sampling period.

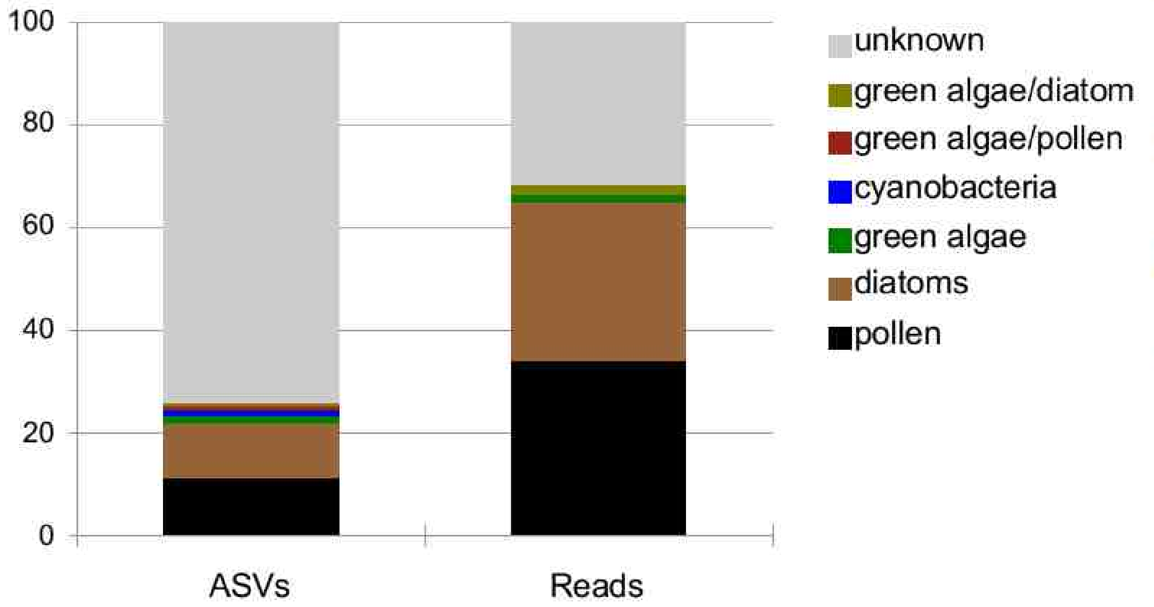

Fig. S5 Proportion of zoosporic fungal ASVs and number of reads according to their substrate association.

Summary statistics alpha diversity (only zoosporic fungi):

Analysis of variance statistics showing the effect of season on zoosporic fungal diversity:

	Sum of sqrs	df	Mean square	F	Pr (>F)
Between groups:	4.97544	4	1.24386	11.8	1.60E-05
					Permutation
Within groups:	2.63484	25	0.10539		p (n=99999)
Total:	7.61028	29	1.00E-05		

Tukey multiple comparison between seasons with Bonferonni corrected p-values:

	diff	lwr	upr	p adj
spring 2015-autumn	-0.61140000	-1.3076920	0.0848920	0.1051880
**spring 2016-autumn**	-0.99530909	-1.5095548	-0.4810634	**0.0000587**
summer -autumn	-0.99530909	-0.6931928	0.3938928	0.9255241
winter -autumn	-0.59506667	-1.2913587	0.1012253	0.1203801
spring 2016-spring 2015	-0.38390909	-1.0049198	0.2371016	0.3873013
summer -spring 2015	0.46175000	-0.1837300	1.1072300	0.2509774
winter -spring 2015	0.01633333	-0.7621448	0.7948115	0.9999965
**summer -spring 2016**	0.84565909	0.4026348	1.2886834	**0.0000715**
winter -spring 2016	0.40024242	-0.2207683	1.0212531	0.3468605
winter -summer	-0.44541667	-1.0908966	0.2000633	0.2829237

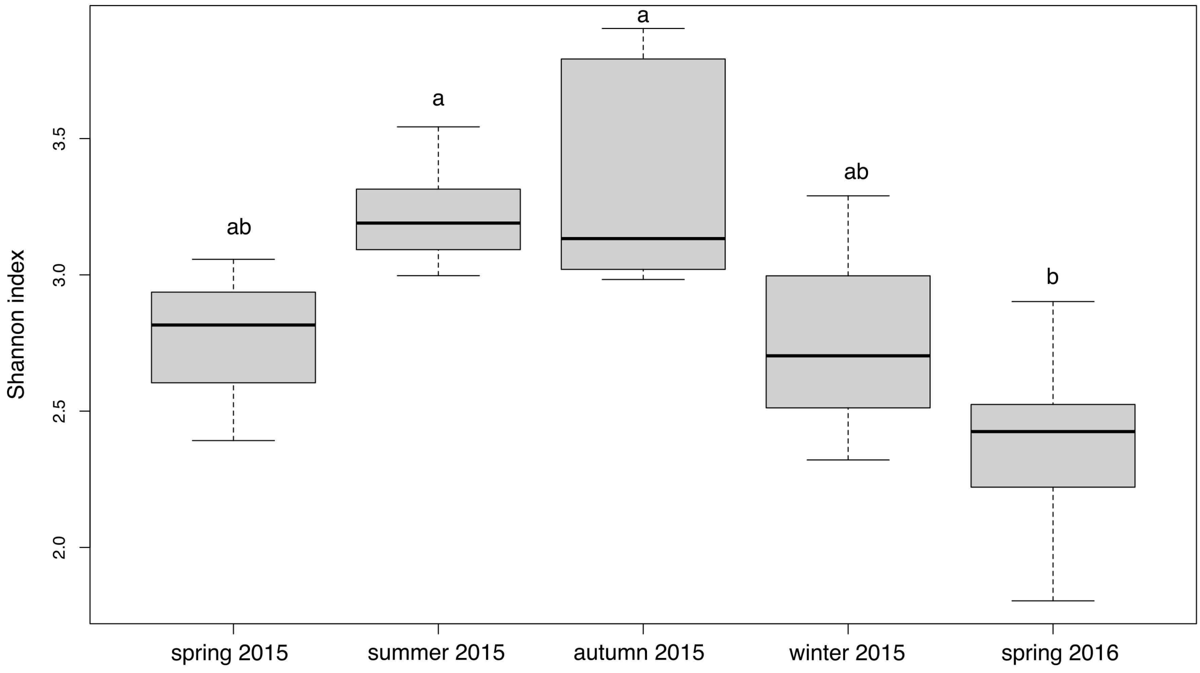

Fig. S6 Seasonal differences in zoosporic fungal diversity (Shannon index). Different letters (a, b) represent significant differences while similar letters (a, a) represent non-significant differences.

Summary statistics beta diversity

PERMANOVA Bray-Curtis

Permutation N:	9999
Total sum of squares:	12.81
Within-group sum of squares:	9387
F:	2.28
p (same):	**0.0001**

Multiple pairwise comparison results after Permanova showing the effect of season on zoosporic fungal community structure.

Bonferroni corrected p-values

	spring 2015	summer 2015	autumn 2015	winter 2015/2016	spring 2016
spring 2015		0.317	0.184	1	1
summer 2015	0.317		**0.012**	*0.064*	**0.003**
autumn 2015	0.184	0.012		0.17	**0.007**
winter 2015/2016	1	0.064	0.17		0.342
spring 2016	1	**0.003**	**0.007**	0.342	

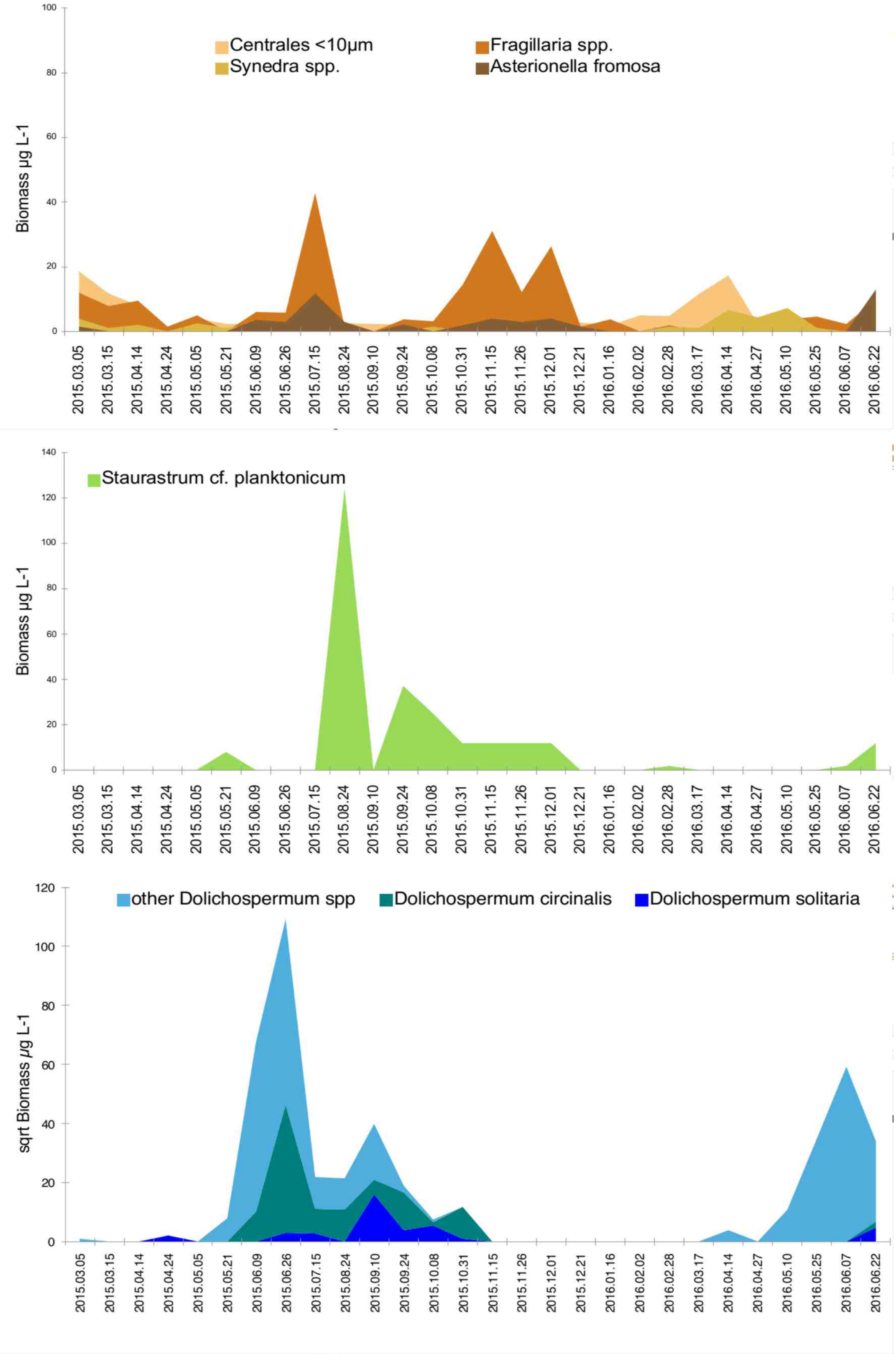

Fig. S7 Temporal dynamics of biomass of the most abundant diatom (upper panel), green algae (middle), and cyanobacteria (lower pannel) host species during the study period (March 2015-June 2016) in Lake Stechlin.

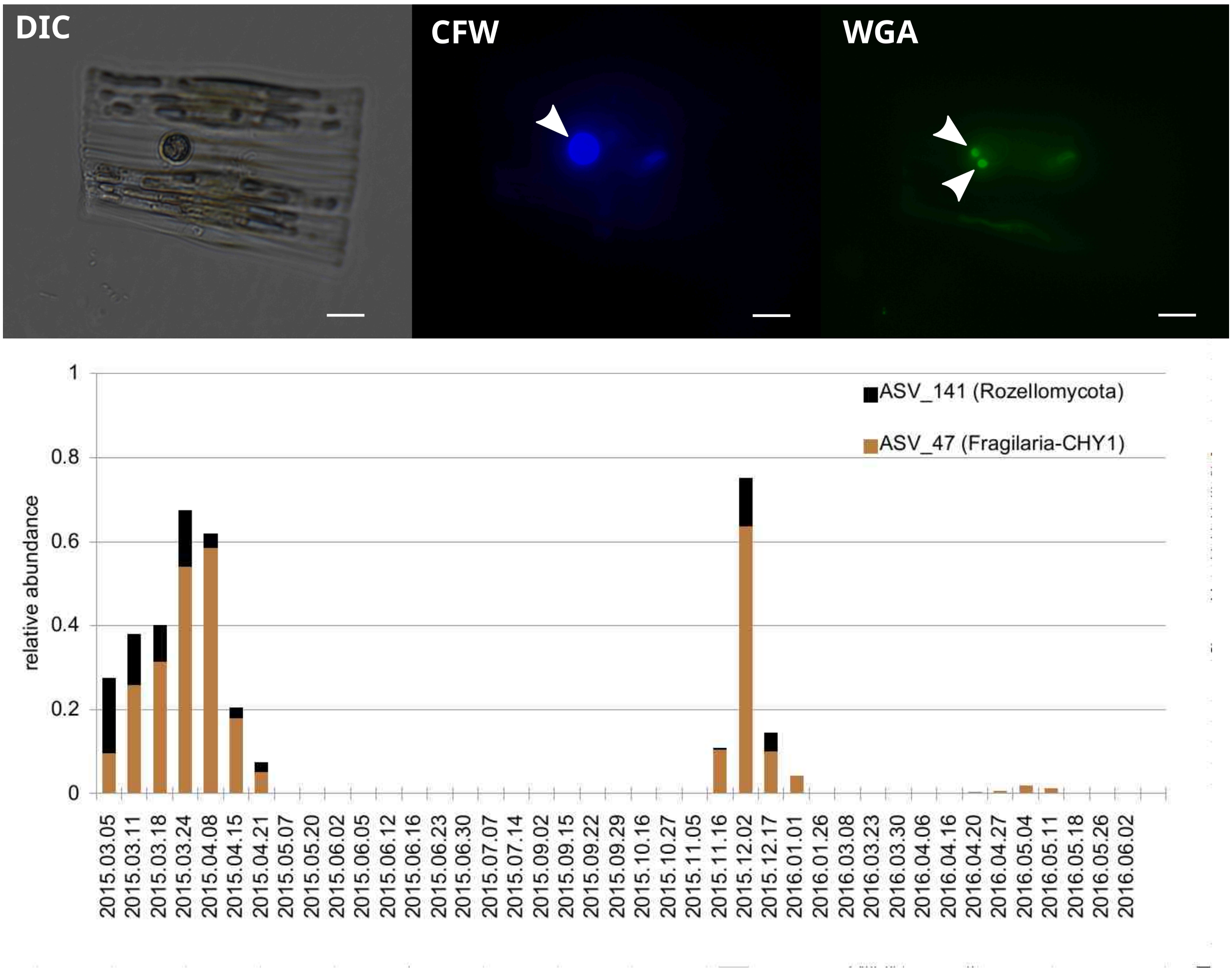

Fig. S8 Above: Microscopy image of *Fragilaria* (diatom) infected by a chytrid which is itself infected by a putative hyperparasite, observed during spring 2015. Chytrid sporangium is visualized in blue with CFW (Calcofluor White) staining and the putative cysts of a Rozella hyperparasite are visualized in green with WGA (Wheat Germ Agglutinin Alexa Fluor 488 Conjugate) staining. DIC - differential interference contrast. Scale bar = 10µm. Below: Temporal co-occurence of chytrid ASV47 (specialist Fragilaria-CHY1) and Rozellomycota ASV141 (Pearson´s $R = 0.97$, $p < 0.001$).

References

1. Padisák J, Scheffler W, Kasprzak P, Koschel R, Krienitz L. Interannual variability in the phytoplankton composition of Lake Stechlin (1994-2000). *Arch FÜR Hydrobiol Beih Adv Limnol* 2003; **58**: 135–155.
2. Rösel S, Rychła A, Wurzbacher C, Grossart H-P. Effects of pollen leaching and microbial degradation on organic carbon and nutrient availability in lake water. *Aquat Sci* 2012; **74**: 87–99.
3. Hillebrand H, Dürselen C-D, Kirschtel D, Pollingher U, Zohary T. Biovolume Calculation for Pelagic and Benthic Microalgae. *J Phycol* 1999; **35**: 403–424.
4. Gruber-Vodicka HR, Seah BKB, Pruesse E. phyloFlash: Rapid Small-Subunit rRNA Profiling and Targeted Assembly from Metagenomes. *mSystems* ; **5**: e00920-20.
5. Stamatakis A. RAxML version 8: a tool for phylogenetic analysis and post-analysis of large phylogenies. *Bioinformatics* 2014; **30**: 1312–1313.
6. Miller MA, Schwartz T, Pickett BE, He S, Klem EB, Scheuermann RH, et al. A RESTful API for Access to Phylogenetic Tools via the CIPRES Science Gateway. *Evol Bioinforma Online* 2015; **11**: 43–48.
7. Tedersoo L, Bahram M, Puusepp R, Nilsson RH, James TY. Novel soil-inhabiting clades fill gaps in the fungal tree of life. *Microbiome* 2017; **5**: 42.
8. Katoh K, Standley DM. MAFFT multiple sequence alignment software version 7: improvements in performance and usability. *Mol Biol Evol* 2013; **30**: 772–780.
9. Capella-Gutiérrez S, Silla-Martínez JM, Gabaldón T. trimAl: a tool for automated alignment trimming in large-scale phylogenetic analyses. *Bioinforma Oxf Engl* 2009; **25**: 1972–1973.
10. Trifinopoulos J, Nguyen L-T, von Haeseler A, Minh BQ. W-IQ-TREE: a fast online phylogenetic tool for maximum likelihood analysis. *Nucleic Acids Res* 2016; **44**: W232–W235.
11. Minh BQ, Nguyen MAT, von Haeseler A. Ultrafast Approximation for Phylogenetic Bootstrap. *Mol Biol Evol* 2013; **30**: 1188–1195.
12. Lepère C; D. Diversity, spatial distribution and activity of fungi in freshwater ecosystems. *PeerJ* 2019; **7**: e6247.
13. Khomich M, Davey ML, Kauserud H, Rasconi S, Andersen T. Fungal communities in Scandinavian lakes along a longitudinal gradient. *Fungal Ecol* 2017; **27**: 36–46.
14. Shearer CA; R. The molecular phylogeny of freshwater Dothideomycetes. *Stud Mycol* 2009; **64**: 145-153S4.
15. Riley R, Salamov AA, Brown DW, Nagy LG, Floudas D, Held BW, et al. Extensive sampling of basidiomycete genomes demonstrates inadequacy of the white-rot/brown-rot paradigm for wood decay fungi. *Proc Natl Acad Sci* 2014; **111**: 9923–9928.
16. Taylor JD, Cunliffe M. Multi-year assessment of coastal planktonic fungi reveals environmental drivers of diversity and abundance. *ISME J* 2016; **10**: 2118–2128.
17. Voronin LV. Terrigenous micromycetes in freshwater ecosystems (review). *Inland Water Biol* 2014; **7**: 352–356.
18. Wurzbacher C, Bärlocher F, Grossart H. Fungi in lake ecosystems. *Aquat Microb Ecol* 2010; **59**: 125–149.
19. Wurzbacher C, Warthmann N, Bourne E, Attermeyer K, Allgaier M, Powell JR, et al. High habitat-specificity in fungal communities in oligo-mesotrophic, temperate Lake Stechlin (North-East Germany). *MycoKeys* 2016; **16**: 17–44.
