# Supplementary figures and images for: Seasonality of parasitic and saprotrophic zoosporic fungi: linking sequence data to ecological traits
Source: ISME J. 2022 Jun 28;16(9):2242–54. doi: 10.1038/s41396-022-01267-y (PMC9381765; doi:10.1038/s41396-022-01267-y)

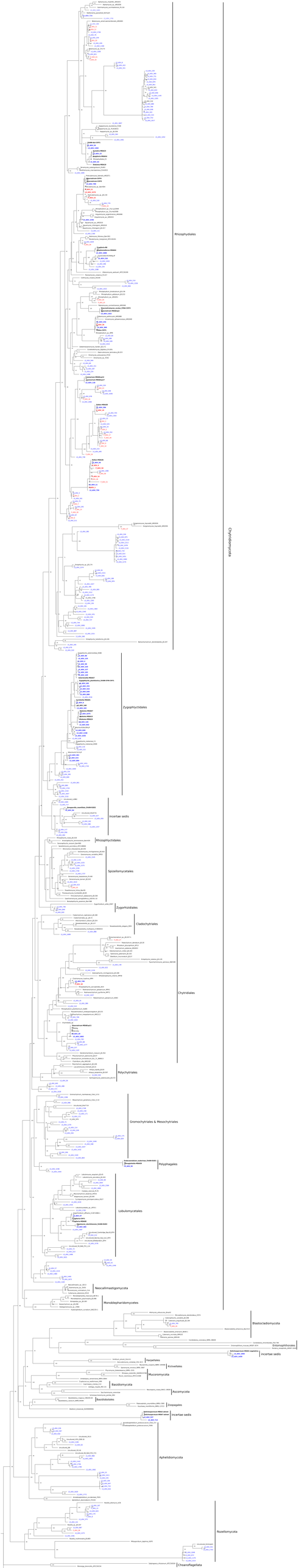

Supplement: Supplementary file 2 — Supplementary figure S9 [file 41396_2022_1267_MOESM2_ESM.pdf]
